# Small-molecule inhibitors of carboxylesterase Notum

**DOI:** 10.4155/fmc-2021-0036

**Published:** 2021-04-22

**Authors:** Yuguang Zhao, Sarah Jolly, Stefano Benvegnu, E Yvonne Jones, Paul V Fish

**Affiliations:** 1 Division of Structural Biology, Wellcome Centre for Human Genetics, University of Oxford, Henry Wellcome Building for Genomic Medicine, Roosevelt Drive, Oxford, OX3 7BN, UK; 2 Alzheimer's Research UK UCL Drug Discovery Institute, University College London, Cruciform Building, Gower Street, London, WC1E 6BT, UK

**Keywords:** activity-based protein profiling, fragment screening, high-throughput screening, hit-finding strategies, Notum inhibitors, structural biology, virtual screening, Wnt signaling

## Abstract

Notum has recently been identified as a negative regulator of Wnt signaling through the removal of an essential palmitoleate group from Wnt proteins. There are emerging reports that Notum plays a role in human disease, with published data suggesting that targeting Notum could represent a new therapeutic approach for treating cancer, osteoporosis and neurodegenerative disorders. Complementary hit-finding strategies have been applied with successful approaches that include high-throughput screening, activity-based protein profiling, screening of fragment libraries and virtual screening campaigns. Structural studies are accelerating the discovery of new inhibitors of Notum. Three fit-for-purpose examples are LP-922056, ABC99 and ARUK3001185. The application of these small-molecule inhibitors is helping to further advance an understanding of the role Notum plays in human disease.

The Wnt signaling pathway, a highly conserved signaling system, controls crucial cellular functions in many aspects of developing and adult mammalian biology [1]. The Wnt pathway transduces signals into a cell through proteins (Wnts) that are secreted as palmitoylated glycoproteins, and the palmitoleoylation state is essential for secreted Wnts to signal [2]. The binding of Wnts to cell surface Frizzled-LRP 5/6 co-receptors activates distinct intracellular cascades, commonly referred to as canonical (β-catenin-dependent) and noncanonical (β-catenin-independent) pathways. The activity, secretion and diffusion of Wnts need, therefore, to be closely regulated to control the downstream signaling. These pathways are tightly regulated by a sophisticated network of modulators and feedbacks, including secreted inhibitory proteins [3] and post-translational modifications [4,5].

There are a number of secreted Wnt inhibitors that prevent Wnt signaling. For example, the Dickkopf family of proteins is composed of secreted Wnt antagonists that bind to LRP 5/6 with high affinity and prevent the ligand–receptor complex formation in response to Wnts [6]. Another group of secreted Wnt inhibitors is the secreted Frizzled-related protein family. These proteins are proposed to antagonize Wnt signaling by directly binding to Wnts with affinities in the nanomolar range, therefore functioning as decoy receptors [7]. Although the common feature among most Wnt inhibitors is blocking Wnt signaling by sterically preventing ligand–receptor interactions or Wnt receptor maturation, a Wnt inhibitor with enzymatic activity called Notum has recently been described [8,9].

## Notum deactivates Wnt signaling

The post-translational acylation of Wnt proteins is regarded as a key step prior to their secretion, transportation and receptor binding [10]. Wnt proteins require *O*-palmitoleoylation of a conserved serine residue (e.g., Ser209 in hWnt3A) as a key post-translational modification for efficient binding to the Frizzled-LRP 5/6 co-receptors, a requirement for signal transduction [11]. The enzyme Porcupine, a member of the membrane-bound *O*-acyl transferase family, attaches palmitoleic acid (**1**) to this serine of Wnt proteins in the endoplasmic reticulum of the cell [12,13]. The development of inhibitors of Porcupine has attracted significant interest as a drug target, with several small-molecule inhibitors entering clinical studies for cancer [14]. An inhibitor of Porcupine would act as an antagonist of Wnt signaling.

Once the Wnt proteins have been palmitoleated, the lipidated Wnts are then secreted and transported to their co-receptors by the carrier protein Wntless and then glypicans (GPC4/6). Delivery from the endoplasmic reticulum to the cellular membrane is performed by Wntless, which is specific for Wnts. However, Wntless does not migrate with the lipidated Wnt into the extracellular space [15]. The glypicans protect the lipid of Wnt proteins in the extracellular aqueous environment and act as a reservoir from which Wnt proteins can be passed to their receptors [16]. Structural studies have recently shown that these carrier proteins also utilize a palmitoleate binding pocket and provide further insight into this mechanism at a molecular level [16,17].

**Figure 1.**
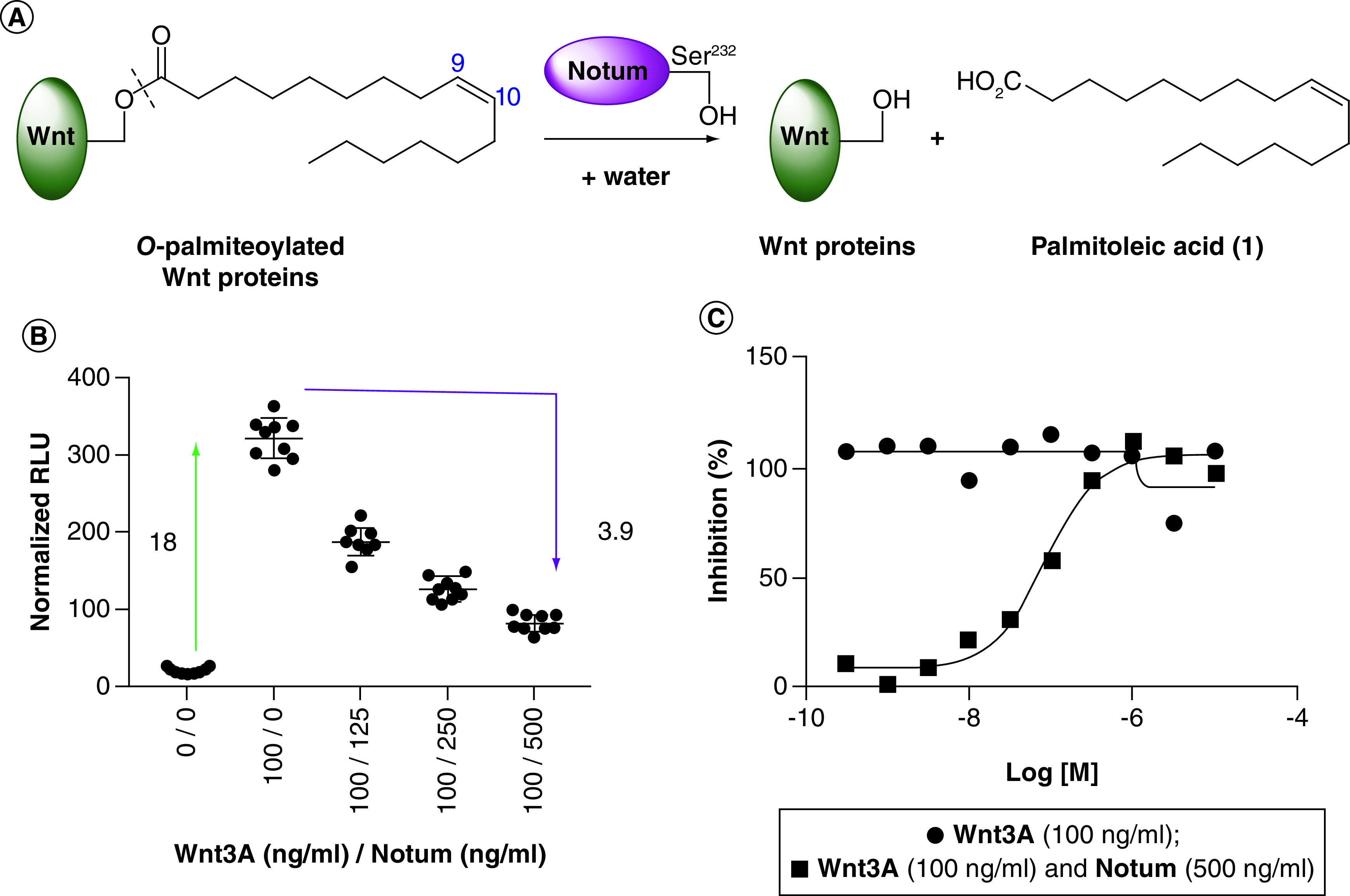
Notum depalmiteoylates Wnt proteins. **(A)** Representation of the biochemical reaction that Notum exerts on palmitoleoylated Wnt proteins. **(B)** A cell-based TCF-LEF reporter assay can be used to assess activity in the Wnt/β-catenin pathway and shows that an increasing concentration of Notum reduces the Wnt3A signaling response. (**C**) Example of concentration–response curves with inhibitor **5** ± Notum. The Wnt3A signaling response is restored in the presence of Notum by the addition of a Notum inhibitor.

Notum was recently identified as an extracellular carboxylesterase that removes the palmitoleate moiety from Wnt proteins, thereby rendering them inactive (Figure 1) [8,9]. Structural analysis revealed the active site in Notum as a large hydrophobic pocket that accommodates the palmitoleate group [8]. The action of Notum upon Wnt signaling with native Wnt substrates can be demonstrated in simple cell-based TCF-LEF reporter assays: an increasing concentration of Notum reduces the Wnt3A signaling response, whereas the Wnt3A response is restored in the presence of Notum by the addition of a Notum inhibitor (Figure 1B & C). It follows that inhibition of Notum could restore Wnt signaling, with potential benefit in disease, where Wnt signaling deficiency is an underlying cause and Notum has been identified as the source.

In addition to the Wnt palmitoleate (C16) substrate, Notum has the potential to hydrolyze shorter lipid-modified proteins. To date, the only other known lipid-modified protein substrate is ghrelin [18]. Notum is able to remove an octanoyl (C8) lipid from ghrelin that is O-linked to Ser3. Thus, modulating the Notum enzyme may offer the potential for regulation of both Wnt signaling and ghrelin-mediated energy homeostasis.

## Notum expression & function

**Figure 2.**
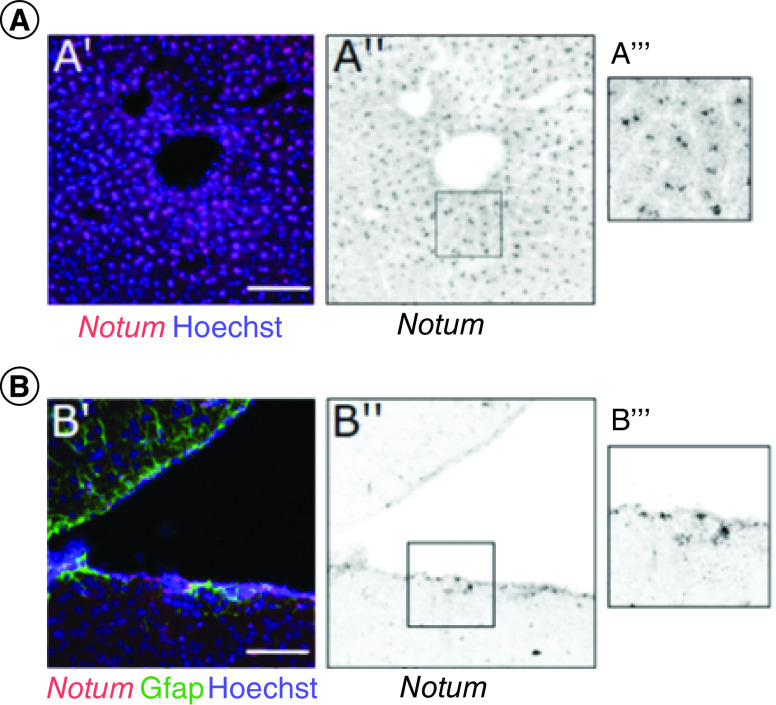
Notum is expressed in the liver and brain in mice. **(A)** RNAscope of Notum (A′ red and A″) showing expression in the liver close to a central vein. A‴ is a zoom image. **(B)** RNAscope of Notum (B′ red and B″) showing expression in the subventricular zone in the brain close to GFAP-positive cells (green). B‴ is a zoom image. Confocal images. Hoechst indicates nuclear staining (blue) in all cases. Scale bar = 100 μm. Courtesy of S Jolly.

Notum has limited peripheral expression, with reports showing its expression in the liver [19], bone [20] and epithelial cells in the intestine [21]. In the brain, Notum has recently been shown to be expressed in the subventricular zone (SVZ) [22], and its expression would be enriched in endothelial cells compared with other brain cell types (Figure 2) [23]. Notum function has been explored using genetic approaches. *Notum*-deleted animals appear normal, and the only phenotypes observed in Notum knockout mice are renal and dental developmental defects [24]. A liver-specific *Notum*-deleted mouse has no phenotype apart from the development of obesity and glucose intolerance with age, and this occurs only in male mice [19].

Because of its extracellular localization and its large hydrophobic active site pocket, Notum has the potential to be targeted by small-molecule inhibitors to restore Wnt signaling in conditions where such a signaling pathway is impaired.

## The emerging role of Notum in disease

Deregulation of the Wnt signaling pathway has been associated with a number of diseases, including cancer, osteoporosis and neurodegenerative disorders such as Alzheimer's disease [25]. We are only beginning to understand the potential role Notum plays in disease, with recent published data suggesting that targeting Notum could represent a potential new therapeutic approach. Genetic and pharmacological approaches have shown that inactivation of Notum leads to an increase in cortical bone mass, suggesting a therapeutic opportunity in osteoporosis [20,26]. In the intestinal crypt, the increased expression of Notum with age leads to a reduction in stem cell maintenance. The application of a small-molecule Notum inhibitor normalizes Wnt signaling and restores epithelial regeneration. Hence, inhibitors of Notum activity may have potential in regenerative medicine [21]. Notum is involved in the progression of colorectal cancer, as Notum expression is increased in human metastatic colorectal cancer cells and proliferation is suppressed by inhibiting the expression of Notum [27]. Finally, it has been recently demonstrated that Notum plays a role in the SVZ of the brain, where it regulates neurogenesis by modulating Wnt signaling [22]. Neural stem cells in the SVZ generate neurons and glial cells throughout life. The researchers identified a neural stem cell intermediate cell population that is enriched for Notum. The secretion of Notum by this subpopulation is proposed to attenuate Wnt-stimulated proliferation in neural stem cell progeny, potentially providing a more favorable environment for the daughter cells. The researchers showed that pharmacological inhibition of Notum leads to an activation of Wnt signaling and increased proliferation in the SVZ. Inhibition of Notum may therefore be a promising therapeutic approach for pathological states where neurogenesis has been shown to be diminished and altered, such as in Alzheimer's disease [28].

There are emerging studies that have used human patient samples to show that Notum levels are changed in disease. Recent work has shown that the *Notum* gene is expressed and upregulated in the brains of Alzheimer's disease patients compared with age-matched controls, although the role of Notum in the mammalian CNS has yet to be established [29]. Notum protein shows a statistically significant difference between peripheral blood samples taken from osteoarthritis patients when compared with healthy individuals [30]. These results suggest that low levels of Notum may contribute to the development of osteoarthritis. An upregulation of Notum was reported in tissue from an animal model of colorectal cancer and human biopsy material [31]. This upregulation of Notum in certain cancers has led to the suggestion that Notum levels in plasma may be a useful pharmacodynamic biomarker of disease [32].

For a molecular target to be druggable, with the potential for translation to the clinic, there will need to be a suitable safety window in the patient population. Pharmacological inhibition of Notum activity could potentially induce proliferation through activation of the Wnt signaling pathway. However, the restricted expression of Notum as well as studies using a global Notum knockout mouse suggests that these risks could be low.

Brommage *et al.* have described the phenotype of a global Notum knockout mouse where the most profound phenotype is that of increased cortical bone thickness and strength; indeed, it was this observation that led them to develop Notum inhibitors as a potential therapeutic for osteoporosis [26]. The global Notum knockout mouse had two developmental phenotypes, dentin dysplasia (tooth malformation) and unilateral kidney agenesis (one kidney), in about a quarter of mice, ascribed to the key role of embryonic Wnt signaling in the development of these tissues. The adult global Notum knockout mouse had slightly reduced body weight, lean body mass and body fat compared with WT mice. Note that this is in complete contrast to the liver-specific Notum knockout, where male mice were reported to be obese [19]. Histological analysis of 40 soft tissues from the Notum global knockout mouse revealed no phenotypic changes. Clinical chemistry and blood cell counts were considered normal, apart from an increase in serum globulins and white blood cell counts, both of which were ascribed to the secondary pulpal and periosteal inflammation associated with the tooth malformation.

Small-molecule Notum inhibitors have been dosed in rodents at pharmacologically relevant doses for up to 18 weeks, and these studies do not report any significant safety issues at this time [22,26]. Although more comprehensive studies on Notum expression and function are still needed, as a whole, data suggest that a context-dependent and targeted inhibition of Notum may open a window on novel therapeutic opportunities and treatment strategies for different pathological states. Ultimately, the safety of inhibiting Notum will need to be evaluated in toxicology studies where on-target/pathway effects have been disengaged from compound-specific toxicity.

## Notum protein structure

**Figure 3.**
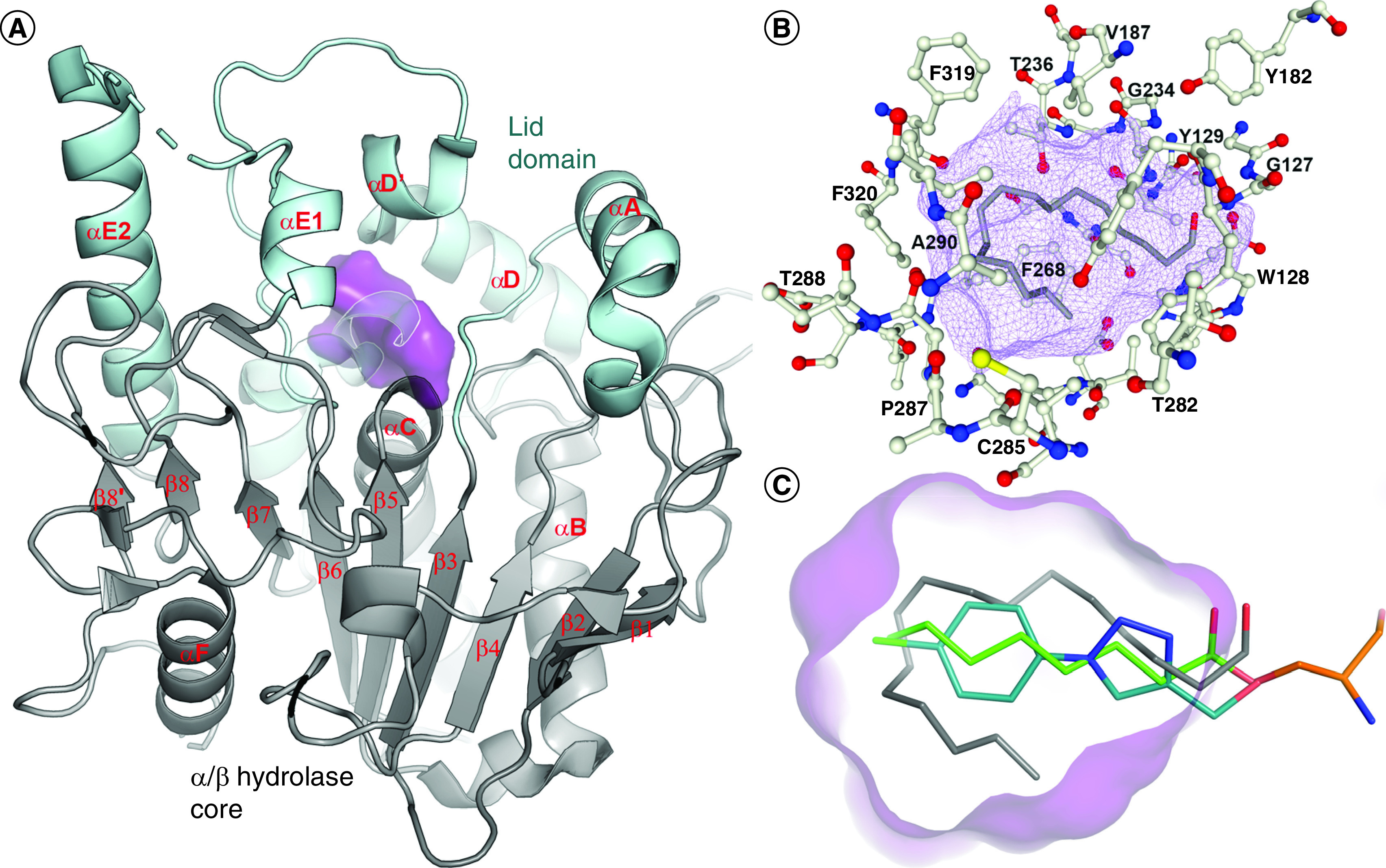
Cartoon representation of Notum structure. **(A)** The enzyme core is shown as a gray cartoon with the lid domain in pale cyan. The lipophilic pocket is outlined as a purple surface. **(B)** Notum pocket-forming residues (white ball and sticks) and the substrate of palmitoleic acid (gray sticks) within the pocket (purple mesh). **(C)** Close-up view of pocket (purple) showing the alignment of substrates of Wnt palmitoleate (gray) and ghrelin octanoyl lipid (green/orange), along with representative inhibitor **15** (teal).

The Notum structure adopts the ‘canonical’ α/β-hydrolase superfamily protein fold, comprising a core domain of eight stranded β-sheets protected by α-helices (αB, αC and αF) and loops. A movable lid domain comprises the αA, αD and αE helices and loops, which can adopt ‘open’ or ‘closed’ conformations by moving the helices away or toward the catalytic pocket, a distinctive feature of lipases (Figure 3A). It is believed that an open state facilitates substrate entry, whereas the closed form is the state for the catalytic processing of substrate. This can be clearly observed with the palmitoleated substrate-bound Notum (S232A) structure, which adopts a closed conformation [8]. By contrast, some small-molecule inhibitors bind to Notum in an open conformation [33].

Notum crystal structures reveal a well-defined, large (approximately 380 Å^3^), hydrophobic active site pocket adjacent to the catalytic triad (Ser232, His389, Asp340) that accommodates the palmitoleate group of Wnt (Protein Data Bank [PDB]: 4UZQ) (Figure 3B) [8]. The hydrophobic binding pocket can accept extended carbon chains up to C8–10, but longer fatty acid chains require a bend in their structure to be accommodated. The oxyanion hole is formed by the canonical Ser232–Ala233 and Gly126–127 amides, along with the Gly127–Trp128 amide, which provides additional stabilization of the tetrahedral transition state during ester hydrolysis. The pocket entrance is comparatively narrow but shows significant flexibility. These structural features have led this pocket in Notum to being assessed as highly druggable [34].

The substrates of Notum and most inhibitors bind within this pocket (Figure 3C). The *cis*-C9–10 double bond of the palmitoleate (C16) tail is positioned at the base of the pocket between Ile291, Phe319 and Phe320, which helps explain the preference for *cis*-unsaturated lipids myristoleic acid (C14) and palmitololeic acid (**1**) (C16) over the* trans*-unsaturated palmitelaidic acid (C16) [8]. The shorter ghrelin octanoyl (C8) lipid adopts a linear conformation in the center of the pocket (PDB: 6ZYF) [18]. There are a number of x-ray structures with small-molecule inhibitors bound to Notum, with most bound in this pocket (e.g., **15** [PDB: 6ZUV]) [35], but with significant variation in position and orientation of the ligand [36].

## Small-molecule inhibitors of Notum

The determination that Notum is a negative regulator of Wnt signaling with a druggable pocket in 2015 has prompted significant efforts to identify small-molecule inhibitors of Notum carboxylesterase activity [36]. There are already a number of inhibitors of Notum (**1–24**) providing a few fit-for-purpose chemical tools (Table 1). Complementary hit-finding strategies have been applied, with successful approaches that include high-throughput screening (HTS), activity-based protein profiling (ABPP), screening of fragment libraries and virtual screening campaigns. Three complementary examples of chemical tools are LP-922056 (**5**), ABC99 (**9**) and ARUK3001185 (**17**), which are suitable for use *in vivo* (Table 2).

**Table 1.** Small-molecule inhibitors of Notum^†^^,^^‡^.

Entry	Hit-finding strategy	Hit	Lead	Comment	Ref.
1	Enzyme biochemical reaction (substrates and products)	**1: palmitoleic acid** IC_50_ 471,000 nM PDB: 4UZQ^§^		• Structural analyses of Notum reveal glycosaminoglycan binding sites and a large hydrophobic pocket at the active site that accommodates palmitoleate. • Saturated C8–12 linear carboxylic acids inhibit activity, whereas longer fatty acids have no effect. • Inhibition was achieved with the Wnt-associated *cis*-unsaturated lipids myristoleic (C14) and palmitoleic (C16) acid but not with *trans*-palmitelaidic acid (C16).	[8]
2	HTS	**2** IC_50_ ND EC_50_ 361 nM	**3: LP-935001** IC_50_ 0.4 nM EC_50_ 12 nM	• Lexicon Pharmaceuticals, Inc. (TX, USA), performed HTS using a cell-based TCF/LEF CellSensor assay. • Structural modifications of hit **2** focused on the tricyclic core, substitution of the aromatic rings and the carboxyl group. • Highly potent inhibitors were identified, including **3**, which has excellent oral bioavailability and systemic half-life in mice. • Compounds **3**, **5** and **6** increased cortical bone thickness and strength in both gonadal intact and ovariectomized rodents by stimulating endocortical bone formation.	[26,37]
3		**4** IC_50_ 2 nM EC_50_ 570 nM	**5: LP-922056** IC_50_ 1 nM EC_50_ 23 nM PDB: 6T2K	• A second lead from Lexicon's HTS was thieno[2,3-d]pyrimidine **4**. • Transposition of the thiophene ring and optimization of the substituents identified **5** as a potent inhibitor. • Mouse pharmacokinetic data for **5** (10 mg/kg p.o.): half-life = 8 h; oral bioavailability = 65%. • Retrospective x-ray structure determination with **5** showed the thienopyrimidine group to effectively fill the palmitoleate pocket, and the acid forms a network of H-bonds with backbone residues Trp^128^, Gly^127^ and Ala^233^ and the side chain of His^389^.	[26,38–40]
4	Scaffold hopping	**6: LP-914822** IC_50_ 2 nM EC_50_ 157 nM PDB: 6T2H	**7** IC_50_ 3.9 nM EC_50_ 220 nM	• Researchers at UCL-Oxford-Crick were seeking to identify a CNS penetrant inhibitor of Notum for use in models of Alzheimer's disease. • Acid **5** was shown to have negligible BBB permeability from mouse PK experiments (K_p_ <0.01). • X-ray structure determination with **5** and **6** showed significant solvent-exposed space at the mouth of the palmitoleate pocket to accommodate amide derivatives. • Scaffold hopping from **6** to a furano[2,3-d]pyrimidine core combined with a preferred amide derivative identified **7** as a potent inhibitor of Notum, with good plasma exposure and reasonable BBB penetration (K_p_ 0.29).	[39,40]
5	ABPP	**8: ABC28** IC_50_ 677 nM^#^^,^^††^	**9: ABC99** IC_50_ 13 nM^#^^,^^††^ EC_50_ 89 nM **10: ABC101** IC_50_ >10 μM^#^ EC_50_ >5 μM	• Application of ABPP discovered NHH carbamates that potently and selectively inhibit Notum. • Optimized irreversible inhibitor ABC99 (**9**) preserves Wnt signaling in the presence of Notum. • ABC99 showed virtually no cross-reactivity with 64 serine hydrolases quantified by MS-ABPP in SW620-treated cells. Only a partial inhibition of ABHD6 was observed. • ABC99 was converted to ABC99yne as a clickable ABPP probe for the visualization of Notum in biological systems. • ABC101 (**10**) is a structurally related, matched, inactive control. • Notum produced by Paneth cells attenuates regeneration of aged intestinal epithelium, and ABC99 inhibition of Notum in mice enhances the regenerative capacity of aged stem cells. • ABC99 and ABC101 have been used to demonstrate that Notum regulates neurogenesis in the V-SVZ of the adult mouse brain.	[21,22,41]
6	Fragment screening (diversity libraries)	**11** IC_50_ 33,000 nM PDB: 6R8P	**12** IC_50_ 85 nM PDB: 6R8R **13** IC_50_ 32 nM	• A crystallographic fragment screen was performed using the XChem platform at Diamond Light Source (Oxford, UK). Crystals of C-terminal His-tagged Notum (Ser81-Thr451 Cys330Ser) were soaked with the DSi-Poised Library (XChem, 768 fragments). • Sixty fragments were observed to bind in the palmitoleate pocket. To date, three of these hits have been disclosed: **11**, **14** and **15**. • Optimization of hit **11** by SAR studies guided by SBDD identified isoquinoline **12** and indazole **13**. • The binding of **12** to Notum was rationalized through an x-ray co-crystal structure determination, which showed a flipped binding orientation compared with hit **11** (Figure 4B).	[42]
7		**14** IC_50_ 37,200 nM PDB: 6TR7		• A second fragment, hit **14**, was highlighted because of its structural similarity to the brain hormone melatonin. • Structural studies were also performed with melatonin and *N*-acetylserotonin. • High-resolution x-ray structures of these complexes showed that two molecules of the ligand bind with Notum.	[33]
8		**15** IC_50_ 11,500 nM PDB: 6ZUV	**16** IC_50_ 18 nM EC_50_ 3500 nM PDB: 6ZVL	• A standout hit from this fragment set was **15** (Figure 3C). • Optimization of **15** by SAR of the heterocyclic head group identified two early lead series as potent inhibitors: 1-phenyl-1,2,3-triazoles and 5-phenyl-1,3,4-oxadiazol-2(3*H*)-ones. • Further investigation of the oxadiazolone series by exploring substitution on the aryl ring, which binds deep in the palmitoleate pocket, identified **16** as a preferred example. • X-ray studies show **16** makes an effective interaction with the oxyanion hole, with hydrogen bonds to three separate amino acids (Gly^127^, Trp^128^, Ala^223^), while still filling the hydrophobic palmitoleate pocket (Figure 4A). • Mouse PK studies with **16** with single oral administration showed good plasma exposure and partial brain penetration (K_p_ 0.16).	[35]
9			**Chemical structure** **not yet disclosed** **17: ARUK3001185** IC_50_ 6.5 nM EC_50_ 110 nM	• Optimization of the 1,2,3-triazole series identified ARUK3001185 (**17**), which was shown to be a potent, selective and brain-penetrant inhibitor of Notum activity suitable for use in both cellular and *in vivo* models of CNS disease.	[35,36,43]
10	Fragment screening (custom-designed libraries)	**18** IC_50_ 6300 nM PDB: 6YV4	**19** IC_50_ 150 nM PDB: 6YXI^‡‡^	• A custom-designed fragment library of carboxylic acids (250 compounds) was screened in a biochemical assay to identify alternative chemical scaffolds as inhibitors of Notum. • A total of 20 hits (criteria: IC_50_ <25 μM; hit rate: 8.0%) were soaked in crystals of Notum, and x-ray structure determination showed 14 fragments bound in the palmitoleate pocket, including **18** and **20**. • Optimization of pyrrole hit **18** by SAR studies guided by SBDD identified 1-phenylpyrrole **19** as the most potent compound from this series.	[44]
11		**20** IC_50_ 48,000 nM PDB: 6YVZ	**21** IC_50_ 110 nM PDB: 6YSK	• Optimization of pyrrolidine hit **20** gave acid (*S*)-**21**. • The 4-chloro-3-(trifluoromethyl)phenyl group was again preferred (*cf.***16**), and a Notum-**21** structure showed the inhibitor fully occupied the palmiteolate pocket while making effective interactions with the oxyanion hole.	[44]
12	Natural products	**22: caffeine** IC_50_ 18,600 nM EC_50_ 45,800 nM K_d_ 85,000 nM^§§^ PDB: 6TV4		• Notum activity can be inhibited by caffeine and, to a lesser degree, theophylline. • The caffeine–Notum interaction was thoroughly characterized by both biochemical and biophysical methods. • High-resolution structures of caffeine and theophylline (**23**, PDB: 6TUZ) show both compounds bind at the center of the palmitoleate pocket but with quite different binding modes (Figure 4C).	[45]
13	Virtual screening	**24** IC_50_ 93 nM^††^ EC_50_ 530 nM PDB: 7ARG^¶¶^		• A virtual screen of the Notum palmiteolate binding pocket was performed by the docking of a curated, virtual chemical library from ChemDiv (CA, USA). • Virtual ‘hits’ with high docking scores were screened in a biochemical assay. Compounds with IC_50_ <500 nM were considered validated experimental hits. • One of these hits was methyl ester **24**. • High-resolution crystal structure of the Notum–inhibitor complex revealed a covalent adduct formed between Ser^232^ and **24** that resembles the substrate acyl-enzyme intermediate (Figure 5). • Covalent inhibition by **24** through the acyl adduct was confirmed by mass spectrometry analysis, which showed an increment of 201.5 Da to the Notum protein following reaction with **24**.	[Steadman D, Atkinson BN, Zhao Y *et al.* Virtual screening directly identifies new drug-like inhibitors of Notum with nanomolar activity. *J. Med. Chem.* (2021), Submitted].

† For a discussion of small-molecule inhibitors of Notum, see [36].

‡ There are several screening assay formats that have been used to identify and characterize inhibitors of Notum. Inhibition of Notum carboxylesterase activity (IC_50_) has been routinely measured in a cell-free biochemical assay with synthetic fluorescent substrates (e.g., OPTS, pNP8). Inhibitors can be screened in cell-based TCF/LEF reporter assays to assess their ability to restore Wnt/β-catenin signaling when activated by an exogenous recombinant Wnt in the presence of Notum. An inhibitor of Notum should show an activation of Wnt signaling (EC_50_) in this model system. IC_50_ and EC_50_ refer to the human Notum OPTS and TCF/LEF assays, respectively, unless stated otherwise.

§ PDB structure of Notum(S232A) with *O*-palmitoleoyl serine.

# Determined by competitive gel-based ABPP.

†† As a covalent inhibitor of Notum, the IC_50_ value will be time-dependent.

‡‡ PDB structure of Notum with 1-(3-chlorophenyl)-2,5-dimethyl-1*H*-pyrrole-3-carboxylic acid.

§§ Determined by SPR.

¶¶ PDB structure of Notum showing S232 acylated by the 4-(indolin-1-yl)-4-oxobutanoyl group.

ABPP: Activity-based protein profiling; BBB: Blood–brain barrier; HTS: High-throughput screening; MS-ABPP: Mass spectrometry activity-based protein profiling; ND: Not determined/disclosed; NHH: *N*-hydroxy-hydantoin; OPTS: Trisodium-8-octanoyloxypyrene; PDB: Protein Data Bank; PK: Pharmacokinetic; pNP8: *p*-nitrophenyl octanoate; p.o.: Orally; SAR: Structure–activity relationship; SBDD: Structure-based drug design; SPR: Surface plasmon resonance; V-SVZ: Ventricular-subventricular zone.

**Table 2.** Comparison of Notum inhibitors LP-922056 (**5**), ABC99 (**9**) and ARUK3001185 (**17**).

Notum–inhibitor structure (PDB)			Chemical structure not yet disclosed
	LP-922056 (5)	ABC99 (9)	ARUK3001185 (17)
	Yes (6T2K)	ND	Yes (ND)
**Notum inhibition**			
OPTS, IC_50_, nM	1.1	170^†^^,^^‡^^,^^§^	6.5
TCF-LEF, EC_50_, nM	23	89	110
**Selectivity**			
Serine hydrolases (number screened)	ND	Yes (64)	Yes (49)
Drug targets (number screened)	ND	ND	Yes (47)
Kinases (number screened)	ND	ND	Yes (485)
**Mouse *in vivo* studies**			
Mouse PK parameters	Yes	No	Yes
Route of administration and dosing regime	3, 10, 30 mg/kg p.o. 25 days	10 mg/kg IP 7 days	2 × 30 mg/kg p.o. b.i.d. 30 days
Brain penetrant (brain:plasma ratio, K_p_)	No (<0.01)	Yes (ND)^#^	Yes (1.08)
Ref.	[26,38,39]	[21,22,41]	[35,36,43]

† Notum IC_50_ data presented for comparison in a common assay format.

‡ As a covalent inhibitor, the IC_50_ value will be time dependent.

§ Competitive gel-based ABPP determined a Notum IC_50_ of 13 nM.

# ABC99 is reported to be a brain penetrant in mice [22].

ABPP: Activity-based protein profiling; b.i.d.: Two-times a day; IP: Intraperitoneal; ND: Not determined/disclosed; PDB: Protein Data Bank; PK: Pharmacokinetic; p.o.: Orally.

The application of a high-throughput screening campaign by Lexicon Pharmaceuticals (TX, USA) identified a number of hits that were optimized to leads suitable for use in rodent models of osteoporosis (Table 1) [37,38]. LP-935001 (**3**), LP-922056 (**5**) and LP-914822 (**6**) were selected as three advanced leads for the program and were used to demonstrate in rodent pharmacology studies, along with complementary approaches, that inhibition of Notum activity is a potential novel anabolic therapy for strengthening cortical bone and preventing nonvertebral fractures [26].

In 2018, Suciu *et al.* described the development of a series of potent and selective irreversible Notum inhibitors discovered using gel-based activity-based protein profiling (Table 1) [41]. Optimized irreversible inhibitor ABC99 (**9**) preserved Wnt signaling in the presence of Notum and showed virtually no cross-reactivity with other serine hydrolases. In addition to ABC99, the researchers described ABC99yne as a clickable activity-based protein profiling probe for the visualization of Notum in biological systems and ABC101 (**10**) as a matched inactive control. These tools have been used to demonstrate that inhibition of Notum in mice enhances the regenerative capacity of aged stem cells [21] and that Notum regulates neurogenesis in the ventricular-subventricular zone of the adult mouse brain [22].

**Figure 4.**
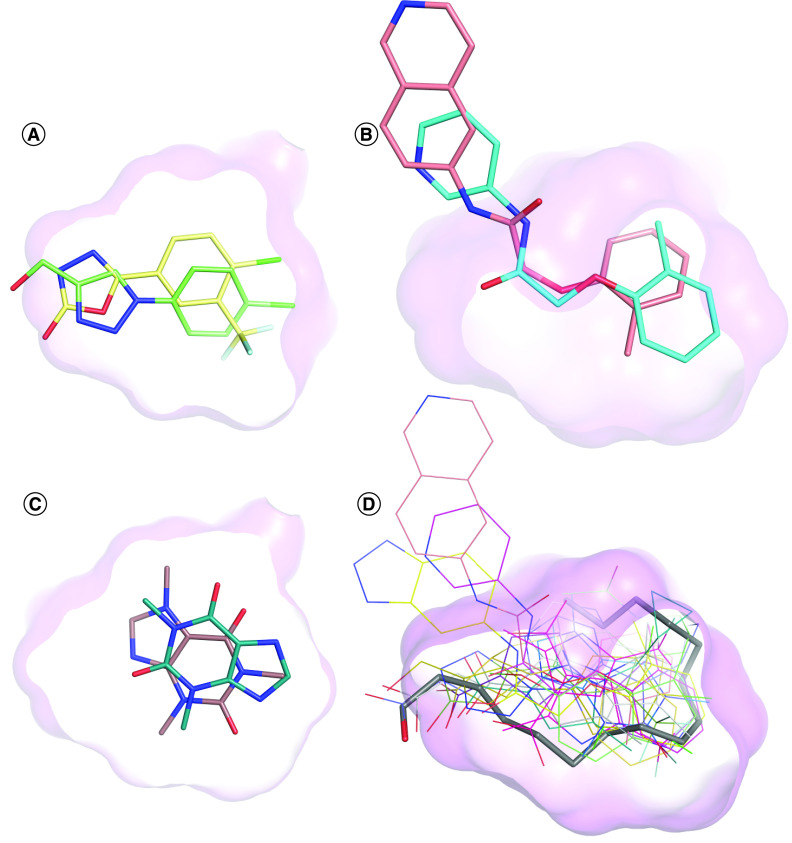
Examples of small-molecule inhibitors of Notum discovered by fragment-based drug design. **(A)** Fragment hit **15** (green, Protein Data Bank [PDB]: 6ZUV) and optimized lead **16** (gold, PDB: 6ZVL). Oxadiazole **16** makes an effective interaction with the oxyanion hole while still filling the pocket (purple). **(B)** Fragment hit **11** (cyan, PDB: 6R8P) and derived inhibitor **12** (salmon, PDB: 6R8R). Isoquinoline **12** shows a flipped binding mode. **(C)** Natural alkaloids caffeine **22** (wheat, PDB: 6TV4) and theophylline **23** (teal, PDB: 6TUZ). Both compounds bind at the center of the pocket but with quite different orientations. **(D)** Overlay of all Notum–inhibitor structures (wire) aligned with *O*-palmitoleate serine (gray, PDB: 4UZQ).

Fragment-based drug design has proven to be an effective strategy for hit-finding across multiple protein target classes [46]. The screening of fragment libraries against Notum's druggable hydrophobic pocket is an attractive strategy and has led to the discovery of a number of hits with orthogonal chemical structures (Figure 4 & Table 1). Fragment screening is performed using both x-ray crystallographic and biochemical platforms in close succession, supported by biophysical screening methods, target occupancy and cell-based TCF/LEF reporter assays [33,35,42,44].

Crystallographic fragment screening technology has significantly advanced in recent years as automated platforms for each step in the process have become available [47]. Zhao and Jones performed a crystallographic fragment screen with Notum using the XChem platform at Diamond Light Source (Oxford, UK) [48]. Crystals of C-terminal His-tagged Notum were soaked with the original DSI-Poised Library (768 fragments), and 60 fragments were found to bind to Notum with a diverse mixture of chemical structures (hit rate: 7.8%); all but one of these hits bound in the hydrophobic pocket of Notum. A standout hit from this screen is 1,2,3-triazole **15** (PDB: 6ZUV) (Figure 4A). Optimization of **15** by structure-based drug design led to the discovery of ARUK3001185 (**17**) as a potent, selective brain-penetrant inhibitor of Notum activity suitable for use in both cellular and *in vivo* models of CNS disease (Table 1) [35,43].

The most recent hit-finding strategy to discover new small-molecule inhibitors of Notum has been the application of virtual screening. There is now an abundance of structural information available for Notum [36], and Steadman *et al. *[steadman d, atkinson bn, zhao y et al. virtual screening directly identifies new drug-like inhibitors of notum with nanomolar activity. j. med. chem. (2021), submitted] performed a virtual screen of the Notum palmiteolate binding pocket by the docking of a curated, virtual chemical library of 534,804 compounds from ChemDiv (CA, USA). Virtual ‘hits’ with high docking scores were purchased and screened in a Notum biochemical assay [steadman d, atkinson bn, zhao y et al. virtual screening directly identifies new drug-like inhibitors of notum with nanomolar activity. j. med. chem. (2021), submitted]. Of the 952 compounds purchased and screened, 31 were found to inhibit Notum (IC_50_ <500 nM) and were considered experimentally validated hits (hit rate: 3.2%); one such hit was methyl 4-(indolin-1-yl)-4-oxobutanoate (**24**) (Table 1) [zhao y, svensson f, steadman d et al. structural insight of notum covalent inhibition, manuscript in preparation]. The application of more traditional hit selection criteria to this data set increased the hit rate to 16.6%, as 158 compounds showed >50% inhibition at 10 μM. These results demonstrate the value of virtual screening with well-trained docking models based on high-resolution structures.

**Figure 5.**
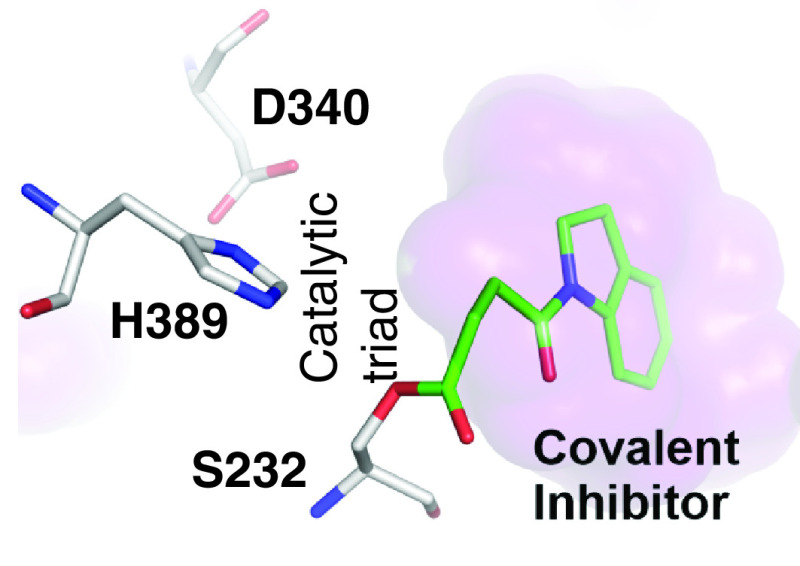
New covalent inhibitor of Notum. High-resolution crystal structure of the Notum–inhibitor complex reveals a covalent adduct formed between Notum and **24**, which resembles the substrate acyl-enzyme intermediate (Protein Data Bank: 7ARG). The indolinyl rings of **24** (green) bind in the pocket (purple surface), whereas the oxobutanoyl chain acylates Ser232 of the catalytic triad (gray).

An x-ray structure of the Notum inhibitor **24** complex revealed that a covalent adduct had formed between the nucleophilic Ser232 of the catalytic triad and the oxobutanoate ester (Figure 5) [zhao y, svensson f, steadman d et al. structural insight of notum covalent inhibition, manuscript in preparation]. The covalent interaction was confirmed by mass spectrometry analysis and was shown not to form with the Notum S232A mutant. Mechanistically, the resulting acyl-enzyme intermediate carbonyl is positioned with an unfavorable angle for the approach of the catalytic water, which, combined with strong hydrophobic interactions with the enzyme pocket residues, hinders the intermediate from being further processed/hydrolyzed and results in covalent inhibition of Notum.

## Future perspective

The definitive identification of Notum as a deactivator of Wnt signaling through the removal of an essential palmitoleate post-translational modification is still a relatively new discovery. Notum can be regarded as a highly druggable enzyme based on de novo analysis of the catalytic pocket, and this has been supported empirically by the independent discovery of a number of quality inhibitors using different hit-finding approaches. Fragment-based and virtual screening approaches have been particularly successful, with high hit rates from multiple screens (3.2–8.0%). Already, a few fit-for-purpose advanced leads have been created from these initial hits and shown utility in rodent models of disease. Hence, it seems likely that new inhibitors of Notum will be discovered, with the potential for optimization to advanced leads. In some respects, it could be argued that this is one of those rare translational research targets where the medicinal chemistry is relatively advanced compared with the understanding of the disease biology, but the two are closely linked. The application of these small-molecule inhibitors is helping to further advance an understanding of the role Notum plays in human disease.

A detailed and comparative description of Notum expression in each body tissue and (within each tissue) across the different cellular types is needed to further understand Notum's role in disease-specific mechanisms of Wnt signaling alterations. Notum is an extracellular secreted enzyme, and so a description of Notum inhibitory activity on Wnt proteins, either as a local signaling molecule or as a more broad Wnt inhibitor, still needs to be addressed. In light of this, Notum interactions with extracellular matrix components, and the identification of structural and functional binding partners in the extracellular milieu, will greatly help in clarifying Notum function.

As summarized in this article, studies in rodent models have shown that inhibiting Notum may have potential in a number of diseases. It seems feasible that additional therapeutic opportunities for Notum inhibitors will be established, especially where Wnt signaling deficiency has been identified as an underlying cause. Realistically, the first Notum inhibitors to enter the clinic will be for the treatment of cancer, with colorectal cancer as a pathfinder indication based on emerging data.

The effective modulation of the Wnt signaling pathway has proven to be challenging, in part because of the lack of druggable molecular targets but also because of on-target toxicity that requires careful management. As these first-generation inhibitors of Notum show efficacy in validated models of disease at credible doses and are then progressed to safety and toxicology studies, it will be possible to establish whether Notum proves to be an effective new drug target in modulating Wnt signaling.

**Executive summary**

**Background**

- The Wnt signaling pathway plays a critical role in both developmental and adult human biology.
- Dysregulation of Wnt signaling has been associated with a number of human diseases.
- Carboxylesterase Notum has been shown to be a negative regulator of Wnt signaling.
- Notum has been shown to be a druggable target for modulating Wnt signaling.

**Notum & disease**

- Inactivation of Notum leads to an increase in cortical bone mass, suggesting a therapeutic opportunity in osteoporosis.
- Notum is involved in the progression of colorectal cancer.
- Inhibition of Notum can promote regeneration of aged tissues.
- Notum plays a role in the subventricular zone of the brain, where it regulates neurogenesis.
- Emerging reports using human patient samples show that Notum levels are changed in diseases such as Alzheimer's disease and osteoarthritis.

**Small-molecule inhibitors of Notum**

- The development of small-molecule inhibitors of Notum is helping to advance an understanding of the role Notum plays in human disease.
- Three leading examples are LP-922056 (**5**), ABC99 (**9**) and ARUK3001185 (**17**).

**Structural biology**

- Structural information has made a significant contribution to our understanding of the mechanism of Notum-mediated hydrolysis of its ester substrates.
- Structural studies are accelerating the discovery of new inhibitors.
- X-ray crystallographic screening of fragment libraries is an effective method of hit discovery that provides rich information for hit-to-lead optimization.
